# The Anaphase-Promoting Complex/Cyclosome Is a Cellular Ageing Regulator

**DOI:** 10.3390/ijms232315327

**Published:** 2022-12-05

**Authors:** Xiangdong Hu, Xuejiao Jin, Xiuling Cao, Beidong Liu

**Affiliations:** 1 State Key Laboratory of Subtropical Silviculture, Zhejiang A&F University, Hangzhou 311300, China; 2 Department of Chemistry and Molecular Biology, University of Gothenburg, 41390 Gothenburg, Sweden

**Keywords:** APC/C, cell cycle, aging, lifespan, cancer, age-related diseases

## Abstract

The anaphase-promoting complex/cyclosome (APC/C) is a complicated cellular component that plays significant roles in regulating the cell cycle process of eukaryotic organisms. The spatiotemporal regulation mechanisms of APC/C in distinct cell cycle transitions are no longer mysterious, and the components of this protein complex are gradually identified and characterized. Given the close relationship between the cell cycle and lifespan, it is urgent to understand the roles of APC/C in lifespan regulation, but this field still seems to have not been systematically summarized. Furthermore, although several reviews have reported the roles of APC/C in cancer, there are still gaps in the summary of its roles in other age-related diseases. In this review, we propose that the APC/C is a novel cellular ageing regulator based on its indispensable role in the regulation of lifespan and its involvement in age-associated diseases. This work provides an extensive review of aspects related to the underlying mechanisms of APC/C in lifespan regulation and how it participates in age-associated diseases. More comprehensive recognition and understanding of the relationship between APC/C and ageing and age-related diseases will increase the development of targeted strategies for human health.

## 1. Introduction

The ability of cells to replicate themselves accurately is crucial to the life and development of all organisms. One of the most important regulatory factors, the anaphase-promoting complex/cyclosome (APC/C), an E3 ubiquitin ligase that specifically targets cell cycle-related proteins for degradation, exhibits essential functions in the regulation of the eukaryotic cell cycle, particularly during anaphase entry and mitotic exit [1,2,3]. The subunits of APC/C are largely conserved from yeast to humans, principally organized into three subcomplexes: the catalytic core (APC2, APC10 and APC11), the tetratricopeptide repeat lobe (APC3, APC6, APC7 and APC8) and the platform (APC1, APC4, APC5 and APC15) subcomplex [4,5]. Two key APC/C activators, Cdc20 and Cdh1, which determine most of its substrate selectivity, control APC/C activity in a cell cycle-dependent manner [6]. In addition, inhibitors, the mitotic checkpoint complex (MCC) and phosphatases interact with the APC/C to spatially and temporally modulate its activity and ensure the accurate execution of mitotic events [7,8,9,10]. During the G1 phase, APC/C^Cdh1^ is an active complex. With the accumulation of G1-cyclins, Cdh1 becomes phosphorylated and separates from the APC/C. This phosphorylation and APC/C^Cdh1^ inactivity will be continued to anaphase [11]. From G2 to prophase, free APC/C is inactivated by its inhibitor Emi1, which associates with Cdc20 and prevents APC/C-Cdc20 binding [7]. At late prophase, Emi1 is proteolyzed, and RASSFA1 takes over the role of this inhibitor until late prometaphase, when the latter is also degraded [12]. Free APC/C is then phosphorylated by Polo-like kinase 1 (Plk1) and cyclin B/cdk1 [13]. At metaphase, APC/C^Cdc20^ is still inactivated owing to the direct binding of the MCC. Once the spindle checkpoint is satisfied, the MCC is separated from APC/C^Cdc20^, and this protein complex achieves its full activity and then induces the proteolysis of securin and cyclin B [3,8]. Degradation of securin actives separase and disassociates sister chromatids from each other by cleaving cohesin complexes, achieving metaphase to anaphase transition [14]. At the same time, continuous cyclin B degradation at anaphase induces dephosphorylation of Cdh1 and a decreased cyclin B activity, which is important for mitotic exit. Meanwhile, Cdc20 is degraded in an APC/C^Cdh1^-dependent manner [3,11]. Thus, the APC/C is a key factor in the regulation of the cell cycle. With a deepening understanding of APC/C, however, it has been widely recognized that APC/C functions include more than mitosis.

Ageing, which is broadly defined as the progressive decline in homeostasis and functional integrity, has attracted great attention and has been a subject of curiosity throughout human history. People have reached some consensus on its hallmarks, such as cellular senescence, genome instability and loss of proteostasis [15,16]. Cellular senescence means the onset of body ageing for multicellular organisms but the end of their reproduction and even death for unicellular organisms. In yeast, various APC/C mutants cause different degrees of cell cycle arrest [17,18,19,20], which is a characteristic of senescent cells [21,22]. APC/C prevents chromosomal aneuploidy by precisely regulating cell cycle progression [23,24], thus, maintaining genomic stability. As a member of the ubiquitin-proteasome system (UPS), the APC/C also plays an important role in the proteostasis network [25]. Additionally, the insufficient function of the APC/C has been observed in ageing-related disease models, including Alzheimer’s disease (AD), premature ageing and cancer models [26,27,28]. Thus, the APC/C seems to have a great relevance with ageing.

This article focuses on the relationship between the APC/C and cellular senescence and reviews recent discoveries that have provided new insights into the potential regulatory mechanisms of the APC/C in ageing and its role in ageing-related diseases.

## 2. The Normal Operation of the APC/C Is Crucial to the Lifespan

On the basis of comprehensive knowledge of this unicellular organism and advanced tools for research into its physiology, budding yeast has become an ideal model organism to study ageing mechanisms in recent years. Sirtuins, mTOR signalling and dietary restrictions are considered key conserved longevity components from yeast to vertebrates [29,30,31]. Moreover, many cellular ageing regulators remain to be discovered.

The APC/C shows great relevance to genome instability and cancer [23], revealing a potential role for APC/C in ageing. In fact, deficient APC/C subunits (*apc5^CA^*, *apc9∆*, *apc10∆* or *cdc26∆*) led to various degrees of shortened replicative lifespan (RLS) in yeast [32]. The *apc10∆* mutant caused the most serious lifespan defect, probably due to its crucial role in the catalytic core and substrate recognition of the APC/C [33]. Interestingly, the lifespan of the *apc5^CA^ apc10∆* double mutant was shorter than either the *apc5^CA^* or *apc10∆* mutant alone, and overexpression of APC5 reduced yeast lifespan [32]. Apc5p is a strict stoichiometric component of the APC/C since reduced or elevated levels of Apc5p were found to reduce the yeast lifespan.

In addition to yeast, APC/C deficiency exacerbates ageing in other species. For instance, the absence of CDC26 destroyed the human oocyte maturation process and led to oocyte ageing, while these defects were partially rescued by overexpression of Cdc26p [34]. Mice lacking Cdh1 entered replicative ageing prematurely due to the stabilization of Ets2 and subsequent activation of *p16^Ink4a^* expression and caused early lethality, revealing an essential role for APC/C in maintaining the RLS of murine embryonic fibroblasts [35]. Interestingly, abnormal activation of the APC/C in mammalian cells also induced ageing, similar to its effect on yeast reported in a previous study [32]. Kuo et al. found that premature activation of the APC/C by T-lymphotropic virus type 1 Tax induced rapid senescence independent of pRb or p53 activity [36]. Mitosis skipping mediated by the p53-dependent premature activation of APC/C^Cdh1^ was necessary and sufficient for senescence induction [37]. Moreover, loss of Emi1-dependent APC/C inhibition elicited DNA damage-induced senescence [38]. Cdh1 is essential for the functions of APC/C in neuronal survival [39] and is tightly regulated by its own degradation, which depends upon two RXXL-type destruction boxes [40]. In human cells, the APC/C inhibitor MAD2L2 sequesters Cdh1 to prevent premature APC/C activation prior to anaphase onset, thereby contributing to mitotic fidelity [41]. Taken together, these studies showed that the normal function of the APC/C is of great significance in cellular senescence from yeasts to humans.

Cell differentiation is an essential process for the growth, development, reproduction, and longevity of all multicellular organisms [42]. The APC/C is also involved in regulating this process by mediating cell cycle withdrawal and promoting certain differentiation-related license factors synthesis [43]. By degradation of Skp2 to stabilize p27, which in turn downregulates Cdks activities, APC/C^Cdh1^ elongates the G1 phase or G0 arrest to coordinate cell type-specific differentiation processes [44]. In response to TGF-β (transforming growth factor beta) stimulation, Smad3 can recruit APC/C^Cdh1^ to ubiquitinate SnoN (Ski-related novel protein N), leading to its degradation and activation of TGF-β target genes and growth inhibition [45]. APC/C^Cdh1^ also targets Id (inhibitor of differentiation/DNA binding) proteins, leading to activation of bHLH (basic helix-loop-helix) transcription factors and its target gene expression, which mediate differentiation in various cell types [46]. However, how the cell cycle and cell cycle-independent functions of APC/C are regulated during development remains poorly understood.

## 3. Mechanisms of the APC/C in Regulating Lifespan

**Figure 1:**
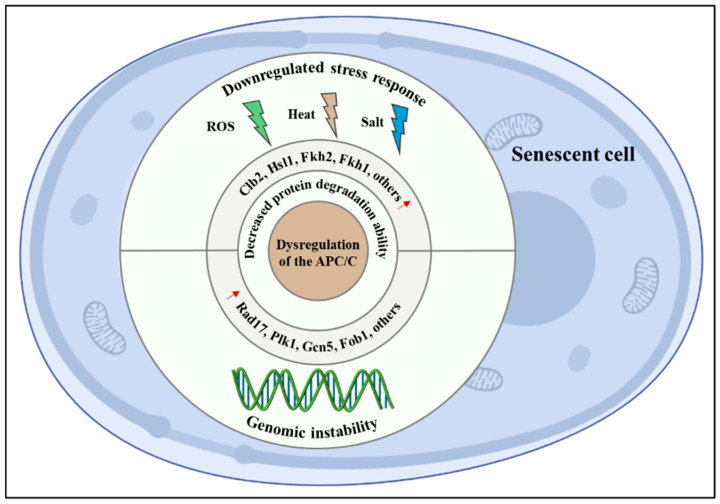
Mechanisms of the anaphase-promoting complex/cyclosome (APC/C) in regulating lifespan. Dysregulation of APC/C can trigger the abnormal accumulation of its substrates, thus, leading to genomic instability and downregulated stress response. Some lifespan determinants, such as Fob1 and Fkh1, are both identified as a bona fide APC/C substrate, indicating that APC/C promotes longevity at least partly owing to the degradation of them.

To our knowledge, the APC/C regulates lifespan through at least the following two mechanisms: maintaining genomic stability and regulating the stress response (Figure 1).

### 3.1. Maintaining Genomic Stability

Genomic instability is a commonly accepted feature of ageing [15]. During normal ageing, genome stability and integrity are continuously challenged by numerous endogenous genotoxins, including DNA replication errors, reactive oxygen species and spontaneous hydrolytic reactions, as well as exogenous threats, such as chemical, physical, and biological agents [47]. Organisms have evolved a complex network of DNA repair mechanisms to deal with this damage collectively [48], but these repair mechanisms appear to be defective during cellular ageing [49]. Some premature ageing syndromes, such as Bloom syndrome and Werner syndrome, have been reported to be related to DNA damage accumulation [50]. In addition, genomic instability is associated with nuclear lamina deficits and aged mitochondrial DNA mutations [51].

Studies have shown that Cdh1 deficiency in mammalian cells caused genomic instability, such as structural and numerical chromosomal aberrations in mouse embryonic fibroblasts and chromosome separation and cytokinesis aberrations in primary human cells [52,53]. However, the exact causes of these phenotypes are unclear. Recently, several protein regulators of genomic stability and DNA damage repairs, such as Rad17, ubiquitin-specific protease 1, and Claspin, as well as the proteins G9a and Glucagon-like peptide, have been shown to be *bona fide* APC/C^Cdh1^ substrates [54,55,56,57]. While Cdh1 overexpression improves emotion and cognitive-related behaviours in global cerebral ischemia rats, indicating that Cdh1 abundance exerts a neuroprotective effect [58]. Endoreduplication is a process of nuclear genome replication in the absence of mitosis, which leads to elevated nuclear gene content and polyploidy [59]. APC/C^Cdh1^ is re-activated after the S phase resulting in reduced Cdk activity, thereby mediating the transition of mitotic cycles to endoreduplication cycles [60]. Altering SlCCS52A (ortholog of Cdh1 in plants) expression in either a positive or negative manner impacts the extent of endoreduplication in fruit and affects fruit size [61]. MAD2L2 plays an important role in several processes, such as DNA double-strand break repair, translesion synthesis and mitosis [62,63]. It is not only the APC/C regulator but also the substrate, which is rapidly degraded by APC/C^Cdc20^ at the onset of anaphase, releasing Cdh1 to activate the dephosphorylated APC/C [41]. Therefore, it is likely that more unidentified APC/C substrates play roles in DNA damage repair and genome integrity, further underlining the importance and relevance of APC/C in maintaining cellular genomic stability.

In addition, activation of APC/C^Cdh1^ was significant to the DNA damage-induced G2 checkpoint in chicken cells [64] and later observed in human cells [56]. Although APC/C^Cdh1^ was active only during the G1 phase and mitotic exit, it was reactivated in the G2 phase in response to genotoxic stress to target the mitotic kinase Plk1, which shows clear potential for facilitating aberrant chromosome separation and DNA replication to delay degradation and prevent mitotic entry until damaged DNA has been repaired [56]. In APC/C^Cdh1^-deficient cells, mitotic entry can still be delayed because of other existing G2-phase DNA damage checkpoints [56], and the APC/C^Cdh1^-dependent checkpoint is not functional in these cells. Thus, cells with impaired DNA may enter mitosis more easily but with more risks, eventually leading to genetic lesions.

Recent studies have suggested that the APC/C plays a role in chromatin assembly and histone modifications [17,65], which are both required for DNA damage repair [66,67]. Based on the genetic interactions between the APC/C with Asf1 and CAF-1 mutants [68], the combinations lead to worsened phenotypes and can be reversed by elevating the expression of Asf1 or any CAF-1 subunits; the APC/C may be involved in DNA damage repair in a chromatin assembly dependent manner. In budding yeast, multiple APC/C subunit mutants showed reduced levels of H3K56^Ac^, H3K9^Ac^ and H3K79^Me^ [65]. H3K56^Ac^ is important for DNA repair and histone deposition [69], H3K9^Ac^ is also required for transcriptional activation [70], and H3K79^Me^ is involved in various activities, such as the regulation of transcription, the cell-cycle checkpoint, DNA repair and cellular development [71]. Thus, the reduced levels of these modifications owing to an impaired APC/C could have a great influence on DNA repair, chromatin and chromosome structure, and transcription. Moreover, the histone acetyltransferase (HAT) GCN5, which is involved in centromere maintenance, DNA repair and transcriptional elongation [72], interacts with the APC/C genetically and functionally and has been shown to be targeted by the APC/C for degradation during the M/G1 transition [65]. Except in yeast, the APC/C has been reported to be involved in the mitotic turnover of TRRAP (TRansformation domain-Associated Protein), a common component of the HAT complex [73].

The molecular mechanisms that determine yeast lifespan have been extensively studied, and one of the important factors has been found to be Fob1 [74,75]. Fob1 antagonistically interacts with Sir2, leading to the accumulation of extrachromosomal ribosomal DNA circles (ERCs) [75], which were the first asymmetrically inherited form of molecular damage identified to cause ageing in yeast. Studies have demonstrated that APC/C plays a role in rDNA silencing, assembly and segregation [76,77]. Recently, Fob1 has been identified as a bona fide APC/C substrate [78]. Deletion of *FOB1* suppressed APC/C-mutant phenotypes, including decreased RLS, increased the rDNA recombination rate and the number of cell cycle defects [78], suggesting that the APC/C maintains genomic stability and, thus, promotes longevity, partially owing, at least, to the degradation of Fob1.

### 3.2. Regulating the Stress Response

Increasing evidence demonstrates that stress, such as genotoxic and oxidative stress, shows a strong relationship with ageing [79,80]. Organisms evolve a series of repair mechanisms to prevent long-term damage, but the balance between the stress response and repair pathways is disrupted during ageing, causing an increased rate of ageing and age-related pathologies [81]. In addition, previous studies have indicated that many of the pathways that modulate stress resistance (such as the PKA, mTOR and Sch9 pathways) also play essential roles in lifespan regulation [31,82].

The Forkhead box (FOX) transcription factor family exhibits a conserved function in the regulation of stress responses and the cell cycle [83]. The APC/C genetically interacts with Fkh proteins, as indicated by the deletion of *FKH1* and *FKH2,* exacerbating the effects of APC/C-mutant phenotypes, such as reduced lifespan and increased oxidative and temperature stress sensitivity, which can be reversed by increasing *FKH* expression [84,85]. Owing to their genetic redundancy, only deletion of both *FKH1* and *FKH2* reduces yeast lifespan in a Forkhead box O (FOXO)-like manner [84]. The Fkh1 protein is degraded specifically during mitosis in a proteasome- and APC/C^Cdc20^-dependent manner, while a stable Fkh1 mutant exhibits increased stress sensitivity and genomic instability and has been associated with a decreased normal lifespan [85]. In fact, targeting of Fox proteins by the APC/C is a conserved process, as indicated by the mammalian Forkhead box M1 (FOXM1) also being identified as a target of the APC/C^Cdh1^ during late mitosis and the early G1 phase for degradation, which is important for regulated entry into the S phase [86].

Several studies have shown that APC/C-defect mutants are sensitive to multiple stresses. In budding yeast, the *cdh1∆* mutant was sensitive to caffeine, ethanol and salt [87], and cells lacking *CDC26* were sensitive to elevated temperature [18]. The accumulation of Hsl1 and Clb2, two APC/C substrates that can disrupt MAPK pathway signalling, induced stress sensitivity, indicating that the APC/C may enhance stress resistance by inhibiting inhibitory signalling [87]. Moreover, the APC/C participates in an acute response to protein-damaging stress by mediating ubiquitination and degradation of heat shock factor 2 (HSF2) and supports cell survival in response to endoplasmic reticulum stress by Cdh1-dependent degradation of its substrates [88,89]. In *Caenorhabditis elegans*, multiple APC/C loss-of-function mutants showed supersensitive phenotype to aldicarb [90], whose responsiveness can be indirectly reflected the muscle activity [91], indicating APC/C can inhibit muscle excitation at the neuromuscular junction.

The list of APC/C substrates continues to increase, as does the number of discoveries showing its involvement in cellular functions. Therefore, the continued study of APC/C substrates involved in regulating lifespan will provide new insight into the role of these substrates in cellular ageing.

## 4. The APC/C in Ageing-Related Diseases

The decline in biological function during ageing is a major trigger for most late-onset diseases, such as cancer, neurodegenerative diseases, cardiovascular disease and diabetes [16,92]. Thus, comprehensive knowledge of the pathogenesis and characteristics of these ageing-related diseases and the development of novel treatment strategies targeting them are very important for life- and healthspan extension.

### 4.1. Neurodegenerative Diseases

Despite various existing clinical manifestations and specifically pathogenic genes, many common neurodegenerative diseases share similar molecular mechanisms. Prototypical examples include Alzheimer’s disease, Huntington’s disease, amyotrophic lateral sclerosis and Parkinson’s disease, all of which contain amyloid inclusion bodies formed by the aggregation of mutated or misfolded proteins during their accumulation because of the diminished capacity of the cellular UPS [93,94]. Considering its role, the UPS is not only a precipitating factor but also a solution, at least in part, in neurodegenerative diseases.

In recent years, emerging evidence has led to the identification of additional novel roles for the APC/C, an E3 ubiquitin ligase of the UPS, in the nervous system and Alzheimer’s disease (AD). Firstly, the APC/C was shown to regulate a series of biological processes in the nervous system, such as axonal morphogenesis [95], neuronal differentiation [96], neuronal cell cycle exit [46], neurogenesis [97], and neuronal survival [35]. TGF-β/Smad2 signalling recruits APC/C^Cdh1^ to degree its substrate SnoN, thus, inhibiting axon growth [95]. Furthermore, two APC/C^Cdh1^ substrates, Id2 and Smurf1, are also involved in axon growth regulation [46,98]. Various APC/C^Cdh1^ substrates have been shown to be involved in the regulation of the cell cycle of neuronal progenitors, such as E2F3A, Ck1 (casein kinase 1) δ and Skp2, whose downregulation is responsible for maintaining the cell cycle exit [99,100,101]. Secondly, the deregulation of APC/C and accumulation of its substrates have been related to AD, as it was shown to be involved in erroneous cell cycle re-entry [102], oxidative stress [103] and excitotoxicity [104]. Cyclin B1 is a cell cycle protein, and it has been shown to accumulate in neurons in AD brains [105]. Maestre et al. reported that Cdk5 phosphorylates Cdh1 and modulates cyclin B1 stability under excitotoxic conditions, thus, inducing neuronal apoptosis in primary culture [102]. Previous studies have shown that there is oxidative damage in AD patients’ brains [106]. The inhibition of Cdh1 causes an increased PFKFB3 (6-phosphofructo-2-kinase/fructose-2,6-bisphosphatase-3) levels, thus, leading to the upregulation of glycolysis. Less glucose is used for the pentose-phosphate pathway, and this causes oxidative stress and apoptosis in neurons [107]. In addition, excitotoxic stimulus stabilizes PFKFB3 by inhibiting APC/C^Cdh1^ and thereby causes neurodegeneration [103]. Compared to healthy individuals in similar age groups, increased glutamate levels are observed in the cerebrospinal fluid of AD patients [108]. Downregulation of APC/C^Cdh1^ causes an accumulation of its substrate glutaminase, which catalyses the conversion of glutamine into glutamate [109]. High levels of glutamate overstimulate the NMDA (*N*-methyl-D-aspartate) receptor, which subsequently leads to an increase in Ca^2^+, thus, causing excitotoxicity and neuronal death [110,111]. Presently, the glutamatergic system is one of the main targets for AD treatment [112]. Finally, it has been reported that oligomeric Aβ, a peptide related to AD, induced proteasome-dependent degradation of cdh1 in vivo in the mouse hippocampus and in vitro in cultured neurons [113]. Furthermore, lower levels of cdh1 have been observed in APP/PS1 mice (an experimental model of AD) compared to age-matched wildtype mice [113]. These studies give strong evidence for the direct involvement of APC/C^Cdh1^ in AD and provide a new clue to explore the pathogenic mechanism and treatment strategy for this neurodegenerative disease.

### 4.2. Cancer

Cancer is one of the leading causes of death worldwide. Although carcinogenesis can occur in any age group, cancer disproportionately jeopardizes individuals 65 years of age and older. The defective operation of the APC/C usually triggers inaccurate mitotic checkpoint signalling, abnormal mitotic exit and uncontrolled genome replication, eventually causing genomic instability, a widely recognized cancer hallmark [114]. Therefore, an increasing number of studies have unsurprisingly identified an essential direct or indirect role for APC/C in cancer.

Deregulation of the APC/C drives oncogenesis. A total of 132 APC/C subunit missense mutations have been identified in cancer, and some of these mutations affect the degree of chromosomal instability, causing cancer cells to adapt during tumour evolution gradually [115]. The expression of APC/C subunits is highly heterogeneous in different cancers. For example, the expression of APC3/CDC27 was significantly elevated in gastric cancer but downregulated in several breast cancer cell lines [116,117]. Interestingly, two APC/C activators play opposing roles in tumorigenesis, with Cdh1 and Cdc20 identified as a tumour suppressor and an oncoprotein, respectively [118]. Downregulation of Cdh1 has been reported in different tumour types, such as prostate, liver, ovary, brain and breast cancers [56,119]. Heterozygous mice lacking Cdh1 showed an increased probability of developing spontaneous tumours [52], further supporting a role for Cdh1 as a tumour suppressor. In contrast to Cdh1, elevated Cdc20 expression has been reported in many cancer cell lines and primary tissues. Overexpression of Cdc20 prevented the inhibition of the spindle assembly checkpoint (SAC) to mediate the APC/C and allowed cell exit from mitosis prematurely [120], eventually leading to genomic instability. Importantly, the ablation of Cdc20 in a mouse model resulted in the efficient regression of skin tumours in vivo [121], confirming the rationale for considering Cdc20 to be an oncoprotein. Moreover, defects in APC/C inhibitory mechanisms led to the occurrence of human diseases, especially cancer [122]. Dysregulation of endogenous APC/C inhibitors, such as Emi1 and Mad2, has been found in various tumour types [123,124]. In addition, recent data also showed that MAD2L2 was significantly upregulated in triple-negative breast cancers and MDA-MB-157 triple-negative cell lines [125].

In addition, many APC/C substrates are implicated in tumorigenesis. The timely destruction of Securin by APC/C is necessary for the transition of metaphase to anaphase during mitosis, and its overexpression can result in aneuploidy [126], which is a hallmark of cancer cells [127]. Securin also serves as a strong prognostic maker in human breast cancer [128], and its accumulation indicates a poor patient outcome [129]. FOXM1 is a member of the FOX family of transcription factors and primarily contributes to the regulation of cell cycle and proliferation [130] and usually accumulates in rapidly dividing cells [131,132]. FOXM1 has achieved great attention for its role in tumour development. Erroneously elevated levels of FOXM1 have been linked to improper cell proliferation, inhibition of apoptotic pathways and cancer metastasis [133,134,135]. In addition, overexpression of FOXM1 is a significant prognostic marker for worsened patient outcomes [136,137]. The Aurora A and B kinases are serine/threonine kinases that are involved in regulating the accurate and equal segregation of genomic material during the cell cycle [138]. Although they have different targets, both of them phosphorylate proteins that promote chromatid segregation during cell division [139]. Elevated levels of Aurora A and B induce chromosomal instability and oncogenesis [107] and have been detected in multiple malignant tumours, such as breast, colorectal and pancreatic cancers [140,141,142]. Overexpression of Aurora A overrides the cell cycle arrest induced by SAC and causes mitotic slippage [143], which is a common phenotype for cancer cells to avoid cell death when treated with mitotic blockers [144]. In addition, the accumulation of Aurora A causes inhibitory phosphorylation of p73, a tumour suppressor with similar functions to p53 [145], further inducing abnormal apoptotic pathways and promoting mitotic slippage [146]. Plk1 is a serine/threonine kinase that plays great roles in the progression and withdrawal of mitosis and is implicated in tumour development [147,148]. In breast cancer, oropharyngeal carcinomas and non-small cell lung cancer, Plk1 is considered a prognostic marker for worsened patient outcomes [149,150,151]. Plk1 depletion in cancer cells induces apoptosis [152], while Plk1 accumulation promotes tumour formation induced by DNA damage [153]. These APC/C substrates have commonly been considered in isolation rather than as a whole population. When combined with the influences of multiple accumulated APC/C substrates on cell biology, such as impaired apoptotic pathways, dysregulation of cell cycle and increased genome instability, the tumour is not far away.

Considering its indispensable role in regulating mitotic progression and tumorigenesis, the APC/C seems to be an attractive therapeutic target for cancer treatment. Apcin and pro-TAME (the prodrug form of tosyl-l-arginine methyl ester) directly inhibited APC/C activity and, thus, were used in combination to suppress tumour cell growth in diverse osteosarcoma and myeloma cancer cell lines [154,155,156]. Many new drugs are being explored to determine their functions as APC/C inhibitors. For example, curcumin has been reported to inhibit pancreatic cell proliferation, probably by downregulating the expression of Cdc20 [157]. Another study showed that a triterpene mixture extracted from the mushroom *Poria cocos* suppressed the migration of pancreatic cancer cells, coinciding with decreased Cdc20 expression [158].

### 4.3. Premature Ageing

Premature ageing syndrome is a rare disease in which certain physiological characteristics associated with normal ageing manifest at an early age; these aberrations include telomere attrition, genome instability and defective stem cell homeostasis during disease development [159,160].

Recent research suggested an unexpected role for the APC/C activator Cdc20 in human premature ageing syndrome [26]. A patient presented with a series of premature ageing phenotypes, including atrophic skin, early hair loss and lack of haematopoietic stem cells, as well as molecular function defects, including SAC failure and chromosomal instability. A de novo heterozygous germline missense mutation, c.856C>A (p. R286S), in *CDC20* was identified by exome sequencing. CDC20 bound to BUBR1 in the formation of the MCC, and APC/C^Cdc20^ activity was inhibited through the MCC. Interestingly, this variant showed decreased binding affinity for BUBR1, but the APC/C^Cdc20^ and MCC interaction was not affected. Moreover, the heterozygous knockout of *CDC20* did not induce SAC failure, but knock-in of mutant *CDC20* induced SAC failure and random aneuploidy in cultured cells, indicating that the pathogenicity of this p. R286S *CDC20* variant could probably be attributed to an imbalance between APC/C^Cdc20^ and MCC activity. This finding associated the APC/C with premature ageing for the first time, but it will not be the last discovery showing this relationship.

## 5. Concluding Remarks and Future Perspectives

In the past few years, significant progress in understanding cell cycle regulation and the indispensable role of APC/C in this process has been achieved. However, only a few of the cell cycle-independent functions of the APC/C in cells have been identified. Therefore, we propose the view that the APC/C is a cellular ageing regulator based on the following points: First, scheduled regulation of the APC/C is crucial to lifespan maintenance. Second, the APC/C is a novel regulator of lifespan, likely because it maintains genomic stability and regulates the stress response. Finally, increasing evidence indicates the direct involvement of APC/C in age-associated diseases, including cancer, AD and premature ageing.

A previous study indicated that the *apc5^CA^ apc10∆* double mutant led to a shorter lifespan than either the *apc5^CA^* or *apc10∆* mutant alone, suggesting that these two subunits probably act in parallel to affect lifespan. To explore this possibility, future work should be directed into investigating whether the APC/C subunits play different roles independent of the APC/C protein complex. Further exploration to determine whether other APC/C subunit mutants can shorten their lifespan is imperative. In particular, the differences in the degree of lifespan reduction due to different gene mutants and double mutants require further investigation. With these results, we will be able to determine whether each APC/C subunit is indispensable for lifespan maintenance and whether subcomplexes affect lifespan independent of the APC/C holoenzyme.

The identification of novel APC/C substrates remains a crucial and active field of research and is essential to advance our knowledge and understanding of its functions, including its regulatory patterns and roles in ageing and related diseases. In many cases, the connection between APC/C and ageing is largely based on the roles of APC/C substrates in lifespan and age-related diseases. Future work should focus on enhancing our understanding of the structural architecture of the APC/C and characterizing the motifs in Cdh1 and Cdc20 critical for substrate-specific recognition and binding. With this information, we can construct a comprehensive APC/C substrate library and classify the substrates according to cellular functions. This work will further expand the APC/C repertoire of known functions to confirm its crucial roles in cellular ageing and other cell cycle-independent processes.

Recent studies have elucidated APC/C functions in the nervous system and DNA damage repair. Diverse protein regulators implicated in DNA damage repair and involved in the maintenance of a stable genome have been identified as APC/C substrates. Not surprisingly, deregulation of the APC/C seems to be associated with AD pathophysiology. Recent evidence has demonstrated that the accumulation of APC/C substrates, such as cyclin B1, PFKFB3, and glutaminase, has significant implications in AD with respect to erroneous cell cycle re-entry, neurodegeneration, oxidative stress and apoptosis in neurons. Furthermore, oligomeric Aβ and glutamate excitotoxicity both reduced Cdh1 levels via proteasome-dependent degradation, inactivating APC/C^Cdh1^ activity with subsequent APC/C substrate accumulation. Based on the similarity of pathogenic mechanisms, it is possible that certain unidentified APC/C substrates accumulate in other neurodegenerative diseases because of deregulated APC/C activity. Future studies to elucidate all the functions of APC/C^Cdc20^ and APC/C^Cdh1^ in diseases and health will be indispensable for determining whether these complexes are valuable therapeutic targets for treating AD and other neurodegenerative diseases.

Increasing evidence indicates that the deregulation of APC/C activity through the mutations of its core subunits or dysregulation of its activators and inhibitors is related to tumorigenesis. A large number of mutations in APC/C subunits have been found in various cancer tissues, and the expression of these mutants is highly heterogeneous. Importantly, two APC/C coactivators play contrasting roles; that is, Cdh1 and Cdc20 function as a tumour suppressor and an oncoprotein, respectively. In fact, Cdh1 can inhibit Cdc20 degradation; thus, APC/C tumorigenic impacts are potential results of imbalanced regulation between these two proteins. Future work should be aimed at elucidating the precise molecular details of carcinogenesis caused by APC/C dysregulation. Further investigation into the causal relations between these APC/C subunits, either individually or collectively and tumorigenesis is also imperative. In addition, accumulating evidence suggests that the APC/C is an attractive therapeutic target for cancer treatment. Future work should be aimed at discovering additional novel potent APC/C inhibitors that can be validated in vivo for designing and developing prospective therapeutic strategies for diverse cancers.

Taken together, APC/C is undoubtedly a novel cellular ageing regulator owing to its significant role in lifespan and age-related diseases. Future work should be aimed at deepening our understanding of the physiological role played by APC/C in these cellular processes, and through the interactions that are confirmed, the repertoire of APC/C functions will be expanded, including those related to its crucial roles in cellular ageing.

## Data Availability

The data presented in this study are available in this paper.

## References

[1] Morgan D.O. (1999). Regulation of the APC and the exit from mitosis. Nat. Cell Biol..

[2] Peters J.M. (2006). The anaphase promoting complex/cyclosome: A machine designed to destroy. Nat. Rev. Mol. Cell Biol..

[3] Sivakumar S., Gorbsky G.J. (2015). Spatiotemporal regulation of the anaphase-promoting complex in mitosis. Nat. Rev. Mol. Cell Biol..

[4] Chang L.F., Zhang Z., Yang J., McLaughlin S.H., Barford D. (2014). Molecular architecture and mechanism of the anaphase-promoting complex. Nature.

[5] Acquaviva C., Pines J. (2006). The anaphase-promoting complex/cyclosome: APC/C. J. Cell Sci..

[6] Visintin R., Prinz S., Amon A. (1997). *CDC20* and *CDH1*: A family of substrate-specific activators of APC-dependent proteolysis. Science.

[7] Reimann J.D., Freed E., Hsu J.Y., Kramer E.R., Peters J.M., Jackson P.K. (2001). Emi1 is a mitotic regulator that interacts with Cdc20 and inhibits the anaphase promoting complex. Cell.

[8] Sudakin V., Chan G.K., Yen T.J. (2001). Checkpoint inhibition of the APC/C in HeLa cells is mediated by a complex of BUBR1, BUB3, CDC20, and MAD2. J. Cell Biol..

[9] Rudner A.D., Murray A.W. (2000). Phosphorylation by Cdc28 activates the Cdc20-dependent activity of the anaphase-promoting complex. J. Cell Biol..

[10] Peters J.M. (2002). The anaphase-promoting complex: Proteolysis in mitosis and beyond. Mol. Cell.

[11] Castro A., Bernis C., Vigneron S., Labbé J.C., Lorca T. (2005). The anaphase-promoting complex: A key factor in the regulation of cell cycle. Oncogene.

[12] Song M.S., Song S.J., Ayad N.G., Chang J.S., Lee J.H., Hong H.K., Lee H., Choi N., Kim J., Kim H. (2004). The tumour suppressor RASSF1A regulates mitosis by inhibiting the APC-Cdc20 complex. Nat. Cell Biol..

[13] Moshe Y., Boulaire J., Pagano M., Hershko A. (2004). Role of Polo-like kinase in the degradation of early mitotic inhibitor 1, a regulator of the anaphase promoting complex/cyclosome. Proc. Natl. Acad. Sci. USA.

[14] Nasmyth K. (1999). Separating sister chromatids. Trends Biochem. Sci..

[15] Lopez-Otin C., Blasco M.A., Partridge L., Serrano M., Kroemer G. (2013). The hallmarks of aging. Cell.

[16] Kennedy B.K., Berger S.L., Brunet A., Campisi J., Cuervo A.M., Epel E.S., Franceschi C., Lithgow G.J., Morimoto R.I., Pessin J.E. (2014). Geroscience: Linking aging to chronic disease. Cell.

[17] Harkness T.A., Davies G.F., Ramaswamy V., Arnason T.G. (2002). The ubiquitin-dependent targeting pathway in *Saccharomyces cerevisiae* plays a critical role in multiple chromatin assembly regulatory steps. Genetics.

[18] Hartwell L.H., Mortimer R.K., Culotti J., Culotti M. (1973). Genetic control of the cell division cycle in yeast: V. genetic analysis of cdc mutants. Genetics.

[19] Heichman K.A., Roberts J.M. (1996). The yeast *CDC16* and *CDC27* genes restrict DNA replication to once per cell cycle. Cell.

[20] Leverson J.D., Joazeiro C.A., Page A.M., Huang H., Hieter P., Hunter T. (2000). The APC11 RING-H2 finger mediates E2-dependent ubiquitination. Mol. Biol. Cell.

[21] Gorgoulis V., Adams P.D., Alimonti A., Bennett D.C., Bischof O., Bishop C., Campisi J., Collado M., Evangelou K., Ferbeyre G. (2019). Cellular senescence: Defining a path forward. Cell.

[22] Hernandez-Segura A., Nehme J., Demaria M. (2018). Hallmarks of cellular senescence. Trends Cell Biol..

[23] Naylor R.M., van Deursen J.M. (2016). Aneuploidy in cancer and aging. Annu. Rev. Genet..

[24] Wang H., Feng K., Wang Q., Deng H. (2021). Reciprocal interaction between SIRT6 and APC/C regulates genomic stability. Sci. Rep..

[25] Labbadia J., Morimoto R.I. (2015). The biology of proteostasis in aging and disease. Annu. Rev. Biochem..

[26] Fujita H., Sasaki T., Miyamoto T., Akutsu S.N., Sato S., Mori T., Nakabayashi K., Hata K., Suzuki H., Kosaki K. (2020). Premature aging syndrome showing random chromosome number instabilities with *CDC20* mutation. Aging Cell.

[27] Fuchsberger T., Lloret A., Vina J. (2017). New functions of APC/C ubiquitin ligase in the nervous system and its role in Alzheimer’s disease. Int. J. Mol. Sci..

[28] Wang Q., Moyret-Lalle C., Couzon F., Surbiguet-Clippe C., Saurin J.C., Lorca T., Navarro C., Puisieux A. (2003). Alterations of anaphase-promoting complex genes in human colon cancer cells. Oncogene.

[29] Wasko B.M., Kaeberlein M. (2014). Yeast replicative aging: A paradigm for defining conserved longevity interventions. FEMS Yeast Res..

[30] Khan A.H., Zou Z., Xiang Y., Chen S., Tian X.L. (2019). Conserved signaling pathways genetically associated with longevity across the species. Biochim. Biophys. Acta Mol. Basis Dis..

[31] Kaeberlein M. (2010). Lessons on longevity from budding yeast. Nature.

[32] Harkness T.A., Shea K.A., Legrand C., Brahmania M., Davies G.F. (2004). A functional analysis reveals dependence on the anaphase-promoting complex for prolonged life span in yeast. Genetics.

[33] Passmore L.A., McCormack E.A., Au S.W., Paul A., Willison K.R., Harper J.W., Barford D. (2003). Doc1 mediates the activity of the anaphase-promoting complex by contributing to substrate recognition. EMBO J..

[34] Li L., Xia Y., Yang Y., Zhang W., Yan H., Yin P., Li K., Chen Y., Lu L., Tong G. (2021). CDC26 is a key factor in human oocyte aging. Hum. Reprod..

[35] Li M., Shin Y.H., Hou L., Huang X., Wei Z., Klann E., Zhang P. (2008). The adaptor protein of the anaphase promoting complex Cdh1 is essential in maintaining replicative lifespan and in learning and memory. Nat. Cell Biol..

[36] Kuo Y.L., Giam C.Z. (2006). Activation of the anaphase promoting complex by HTLV-1 tax leads to senescence. EMBO J..

[37] Johmura Y., Shimada M., Misaki T., Naiki-Ito A., Miyoshi H., Motoyama N., Ohtani N., Hara E., Nakamura M., Morita A. (2014). Necessary and sufficient role for a mitosis skip in senescence induction. Mol. Cell.

[38] Verschuren E.W., Ban K.H., Masek M.A., Lehman N.L., Jackson P.K. (2007). Loss of Emi1-dependent anaphase-promoting complex/cyclosome inhibition deregulates E2F target expression and elicits DNA damage-induced senescence. Mol. Cell Biol..

[39] Almeida A. (2012). Regulation of APC/C-Cdh1 and its function in neuronal survival. Mol. Neurobiol..

[40] Listovsky T., Oren Y.S., Yudkovsky Y., Mahbubani H.M., Weiss A.M., Lebendiker M., Brandeis M. (2004). Mammalian Cdh1/Fzr mediates its own degradation. EMBO J..

[41] Listovsky T., Sale J.E. (2013). Sequestration of CDH1 by MAD2L2 prevents premature APC/C activation prior to anaphase onset. J. Cell Biol..

[42] Sánchez Alvarado A., Yamanaka S. (2014). Rethinking differentiation: Stem cells, regeneration, and plasticity. Cell.

[43] Qiao X., Zhang L., Gamper A.M., Fujita T., Wan Y. (2010). APC/C-Cdh1: From cell cycle to cellular differentiation and genomic integrity. Cell Cycle.

[44] Wei W., Ayad N.G., Wan Y., Zhang G.J., Kirschner M.W., Kaelin W.G. (2004). Degradation of the SCF component Skp2 in cell-cycle phase G1 by the anaphase-promoting complex. Nature.

[45] Stroschein S.L., Bonni S., Wrana J.L., Luo K. (2001). Smad3 recruits the anaphase-promoting complex for ubiquitination and degradation of SnoN. Genes Dev..

[46] Lasorella A., Stegmüller J., Guardavaccaro D., Liu G., Carro M.S., Rothschild G., de la Torre-Ubieta L., Pagano M., Bonni A., Iavarone A. (2006). Degradation of Id2 by the anaphase-promoting complex couples cell cycle exit and axonal growth. Nature.

[47] Hoeijmakers J.H. (2009). DNA damage, aging, and cancer. N. Engl. J. Med..

[48] Lord C.J., Ashworth A. (2012). The DNA damage response and cancer therapy. Nature.

[49] Young T.Z., Liu P., Urbonaite G., Acar M. (2019). Quantitative Insights into Age-Associated DNA-Repair Inefficiency in Single Cells. Cell Rep..

[50] Burtner C.R., Kennedy B.K. (2010). Progeria syndromes and ageing: What is the connection?. Nat. Rev. Mol. Cell Biol..

[51] Kauppila T.E.S., Bratic A., Jensen M.B., Baggio F., Partridge L., Jasper H., Gronke S., Larsson N.G. (2018). Mutations of mitochondrial DNA are not major contributors to aging of fruit flies. Proc. Natl. Acad. Sci. USA.

[52] Garcia-Higuera I., Manchado E., Dubus P., Canamero M., Mendez J., Moreno S., Malumbres M. (2008). Genomic stability and tumour suppression by the APC/C cofactor Cdh1. Nat. Cell Biol..

[53] Engelbert D., Schnerch D., Baumgarten A., Wasch R. (2008). The ubiquitin ligase APC^Cdh1^ is required to maintain genome integrity in primary human cells. Oncogene.

[54] Zhang L., Park C.H., Wu J., Kim H., Liu W., Fujita T., Balasubramani M., Schreiber E.M., Wang X.F., Wan Y. (2010). Proteolysis of Rad17 by Cdh1/APC regulates checkpoint termination and recovery from genotoxic stress. EMBO J..

[55] Cotto-Rios X.M., Jones M.J., Busino L., Pagano M., Huang T.T. (2011). APC/C^Cdh1^-dependent proteolysis of USP1 regulates the response to UV-mediated DNA damage. J. Cell Biol..

[56] Bassermann F., Frescas D., Guardavaccaro D., Busino L., Peschiaroli A., Pagano M. (2008). The Cdc14B-Cdh1-Plk1 axis controls the G2 DNA-damage-response checkpoint. Cell.

[57] Takahashi A., Imai Y., Yamakoshi K., Kuninaka S., Ohtani N., Yoshimoto S., Hori S., Tachibana M., Anderton E., Takeuchi T. (2012). DNA damage signaling triggers degradation of histone methyltransferases through APC/C^Cdh1^ in senescent cells. Mol. Cell.

[58] Zhang B., Chen X., Lv Y., Wu X., Gui L., Zhang Y., Qiu J., Song G., Yao W., Wan L. (2019). Cdh1 overexpression improves emotion and cognitive-related behaviors via regulating hippocampal neuroplasticity in global cerebral ischemia rats. Neurochem. Int..

[59] Larkins B.A., Dilkes B.P., Dante R.A., Coelho C.M., Woo Y.M., Liu Y. (2001). Investigating the hows and whys of DNA endoreduplication. J. Exp. Bot..

[60] Eguren M., Manchado E., Malumbres M. (2011). Non-mitotic functions of the anaphase-promoting complex. Semin. Cell Dev. Biol..

[61] Mathieu-Rivet E., Gévaudant F., Cheniclet C., Hernould M., Chevalier C. (2010). The anaphase promoting complex activator CCS52A, a key factor for fruit growth and endoreduplication in tomato. Plant Signal. Behav..

[62] de Krijger I., Föhr B., Pérez S.H., Vincendeau E., Serrat J., Thouin A.M., Susvirkar V., Lescale C., Paniagua I., Hoekman L. (2021). MAD2L2 dimerization and TRIP13 control shieldin activity in DNA repair. Nat. Commun..

[63] Pernicone N., Grinshpon S., Listovsky T. (2020). CDH1 binds MAD2L2 in a Rev1-like pattern. Biochem. Biophys. Res. Commun..

[64] Sudo T., Ota Y., Kotani S., Nakao M., Takami Y., Takeda S., Saya H. (2001). Activation of Cdh1-dependent APC is required for G1 cell cycle arrest and DNA damage-induced G2 checkpoint in vertebrate cells. EMBO J..

[65] Turner E.L., Malo M.E., Pisclevich M.G., Dash M.D., Davies G.F., Arnason T.G., Harkness T.A. (2010). The *Saccharomyces cerevisiae* anaphase-promoting complex interacts with multiple histone-modifying enzymes to regulate cell cycle progression. Eukaryot Cell.

[66] Linger J.G., Tyler J.K. (2007). Chromatin disassembly and reassembly during DNA repair. Mutat. Res..

[67] Tessarz P., Kouzarides T. (2014). Histone core modifications regulating nucleosome structure and dynamics. Nat. Rev. Mol. Cell Biol..

[68] Kim J.A., Haber J.E. (2009). Chromatin assembly factors Asf1 and CAF-1 have overlapping roles in deactivating the DNA damage checkpoint when DNA repair is complete. Proc. Natl. Acad. Sci. USA.

[69] Rodriges Blanko E., Kadyrova L.Y., Kadyrov F.A. (2016). DNA mismatch repair interacts with CAF-1- and ASF1A-H3-H4-dependent histone (H3-H4)2 tetramer deposition. J. Biol. Chem..

[70] Gates L.A., Shi J., Rohira A.D., Feng Q., Zhu B., Bedford M.T., Sagum C.A., Jung S.Y., Qin J., Tsai M.J. (2017). Acetylation on histone H3 lysine 9 mediates a switch from transcription initiation to elongation. J. Biol. Chem..

[71] Farooq Z., Banday S., Pandita T.K., Altaf M. (2016). The many faces of histone H3K79 methylation. Mutat. Res. Rev. Mutat. Res..

[72] Espinosa M.C., Rehman M.A., Chisamore-Robert P., Jeffery D., Yankulov K. (2010). *GCN5* is a positive regulator of origins of DNA replication in *Saccharomyces cerevisiae*. PLoS ONE.

[73] Ichim G., Mola M., Finkbeiner M.G., Cros M.P., Herceg Z., Hernandez-Vargas H. (2014). The histone acetyltransferase component TRRAP is targeted for destruction during the cell cycle. Oncogene.

[74] Sinclair D.A., Guarente L. (1997). Extrachromosomal rDNA circles—A cause of aging in yeast. Cell.

[75] Defossez P.A., Prusty R., Kaeberlein M., Lin S.J., Ferrigno P., Silver P.A., Keil R.L., Guarente L. (1999). Elimination of replication block protein Fob1 extends the life span of yeast mother cells. Mol. Cell.

[76] Sullivan M., Holt L., Morgan D.O. (2008). Cyclin-specific control of ribosomal DNA segregation. Mol. Cell Biol..

[77] Dubey R.N., Nakwal N., Bisht K.K., Saini A., Haldar S., Singh J. (2009). Interaction of APC/C-E3 ligase with Swi6/HP1 and Clr4/Suv39 in heterochromatin assembly in fission yeast. J. Biol. Chem..

[78] Menzel J., Malo M.E., Chan C., Prusinkiewicz M., Arnason T.G., Harkness T.A. (2014). The anaphase promoting complex regulates yeast lifespan and rDNA stability by targeting Fob1 for degradation. Genetics.

[79] Yousefzadeh M., Henpita C., Vyas R., Soto-Palma C., Robbins P., Niedernhofer L. (2021). DNA damage-how and why we age?. Elife.

[80] Vatner S.F., Zhang J., Oydanich M., Berkman T., Naftalovich R., Vatner D.E. (2020). Healthful aging mediated by inhibition of oxidative stress. Ageing Res. Rev..

[81] Haigis M.C., Yankner B.A. (2010). The aging stress response. Mol. Cell.

[82] Longo V.D., Fabrizio P. (2002). Regulation of longevity and stress resistance: A molecular strategy conserved from yeast to humans?. Cell Mol. Life Sci..

[83] Furukawa-Hibi Y., Kobayashi Y., Chen C., Motoyama N. (2005). FOXO transcription factors in cell-cycle regulation and the response to oxidative stress. Antioxid. Redox Signal..

[84] Postnikoff S.D., Malo M.E., Wong B., Harkness T.A. (2012). The yeast forkhead transcription factors fkh1 and fkh2 regulate lifespan and stress response together with the anaphase-promoting complex. PLoS Genet..

[85] Malo M.E., Postnikoff S.D., Arnason T.G., Harkness T.A. (2016). Mitotic degradation of yeast Fkh1 by the anaphase promoting complex is required for normal longevity, genomic stability and stress resistance. Aging.

[86] Park H.J., Costa R.H., Lau L.F., Tyner A.L., Raychaudhuri P. (2008). Anaphase-promoting complex/cyclosome-CDH1-mediated proteolysis of the forkhead box M1 transcription factor is critical for regulated entry into S phase. Mol. Cell Biol..

[87] Simpson-Lavy K.J., Sajman J., Zenvirth D., Brandeis M. (2014). APC/C^Cdh1^ specific degradation of Hsl1 and Clb2 is required for proper stress responses of *S. cerevisiae*. Cell Cycle.

[88] Ahlskog J.K., Bjork J.K., Elsing A.N., Aspelin C., Kallio M., Roos-Mattjus P., Sistonen L. (2010). Anaphase-promoting complex/cyclosome participates in the acute response to protein-damaging stress. Mol. Cell Biol..

[89] Chen M., Gutierrez G.J., Ronai Z.A. (2012). The anaphase-promoting complex or cyclosome supports cell survival in response to endoplasmic reticulum stress. PLoS ONE.

[90] Kowalski J.R., Dube H., Touroutine D., Rush K.M., Goodwin P.R., Carozza M., Didier Z., Francis M.M., Juo P. (2014). The Anaphase-Promoting Complex (APC) ubiquitin ligase regulates GABA transmission at the *C. elegans* neuromuscular junction. Mol. Cell. Neurosci..

[91] Mahoney T.R., Luo S., Nonet M.L. (2006). Analysis of synaptic transmission in *Caenorhabditis elegans* using an aldicarb-sensitivity assay. Nat. Protoc..

[92] Niccoli T., Partridge L. (2012). Ageing as a risk factor for disease. Curr. Biol..

[93] Ross C.A., Poirier M.A. (2004). Protein aggregation and neurodegenerative disease. Nat. Med..

[94] Dantuma N.P., Bott L.C. (2014). The ubiquitin-proteasome system in neurodegenerative diseases: Precipitating factor, yet part of the solution. Front. Mol. Neurosci..

[95] Stegmuller J., Konishi Y., Huynh M.A., Yuan Z., Dibacco S., Bonni A. (2006). Cell-intrinsic regulation of axonal morphogenesis by the Cdh1-APC target SnoN. Neuron.

[96] Harmey D., Smith A., Simanski S., Moussa C.Z., Ayad N.G. (2009). The anaphase promoting complex induces substrate degradation during neuronal differentiation. J. Biol. Chem..

[97] Delgado-Esteban M., Garcia-Higuera I., Maestre C., Moreno S., Almeida A. (2013). APC/C-Cdh1 coordinates neurogenesis and cortical size during development. Nat. Commun..

[98] Kannan M., Lee S.J., Schwedhelm-Domeyer N., Stegmüller J. (2012). The E3 ligase Cdh1-anaphase promoting complex operates upstream of the E3 ligase Smurf1 in the control of axon growth. Development.

[99] Ping Z., Lim R., Bashir T., Pagano M., Guardavaccaro D. (2012). APC/C (Cdh1) controls the proteasome-mediated degradation of E2F3 during cell cycle exit. Cell Cycle.

[100] Penas C., Govek E.E., Fang Y., Ramachandran V., Daniel M., Wang W., Maloof M.E., Rahaim R.J., Bibian M., Kawauchi D. (2015). Casein kinase 1δ is an APC/C^Cdh1^ substrate that regulates cerebellar granule cell neurogenesis. Cell Rep..

[101] Bashir T., Dorrello N.V., Amador V., Guardavaccaro D., Pagano M. (2004). Control of the SCF(Skp2-Cks1) ubiquitin ligase by the APC/C(Cdh1) ubiquitin ligase. Nature.

[102] Maestre C., Delgado-Esteban M., Gomez-Sanchez J.C., Bolaños J.P., Almeida A. (2008). Cdk5 phosphorylates Cdh1 and modulates cyclin B1 stability in excitotoxicity. EMBO J..

[103] Rodriguez-Rodriguez P., Fernandez E., Almeida A., Bolanos J.P. (2012). Excitotoxic stimulus stabilizes PFKFB3 causing pentose-phosphate pathway to glycolysis switch and neurodegeneration. Cell Death Differ..

[104] Burbaeva G., Boksha I.S., Tereshkina E.B., Savushkina O.K., Starodubtseva L.I., Turishcheva M.S. (2005). Glutamate metabolizing enzymes in prefrontal cortex of Alzheimer’s disease patients. Neurochem. Res..

[105] Vincent I., Jicha G., Rosado M., Dickson D.W. (1997). Aberrant expression of mitotic cdc2/cyclin B1 kinase in degenerating neurons of Alzheimer’s disease brain. J. Neurosci..

[106] Smith M.A., Perry G., Richey P.L., Sayrec L.M., Anderson V.E., Beal M.F., Kowall N. (1996). Oxidative damage in Alzheimer’s. Nature.

[107] Herrero-Mendez A., Almeida A., Fernandez E., Maestre C., Moncada S., Bolanos J.P. (2009). The bioenergetic and antioxidant status of neurons is controlled by continuous degradation of a key glycolytic enzyme by APC/C-Cdh1. Nat. Cell Biol..

[108] Pomara N., Singh R., Deptula D., Chou J.C., Schwartz M.B., LeWitt P.A. (1992). Glutamate and other CSF amino acids in Alzheimer’s disease. Am. J. Psychiatry.

[109] Colombo S.L., Palacios-Callender M., Frakich N., Carcamo S., Kovacs I., Tudzarova S., Moncada S. (2011). Molecular basis for the differential use of glucose and glutamine in cell proliferation as revealed by synchronized HeLa cells. Proc. Natl. Acad. Sci. USA.

[110] Norris C.M., Blalock E.M., Thibault O., Brewer L.D., Clodfelter G.V., Porter N.M., Landfield P.W. (2006). Electrophysiological mechanisms of delayed excitotoxicity: Positive feedback loop between NMDA receptor current and depolarization-mediated glutamate release. J. Neurophysiol..

[111] LaFerla F.M. (2002). Calcium dyshomeostasis and intracellular signalling in Alzheimer’s disease. Nat. Rev. Neurosci..

[112] Zádori D., Veres G., Szalárdy L., Klivényi P., Toldi J., Vécsei L. (2014). Glutamatergic dysfunctioning in Alzheimer’s disease and related therapeutic targets. J. Alzheimer’s Dis..

[113] Fuchsberger T., Martinez-Bellver S., Giraldo E., Teruel-Marti V., Lloret A., Vina J. (2016). Aβ induces excitotoxicity mediated by APC/C-Cdh1 depletion that can be prevented by glutaminase inhibition promoting neuronal survival. Sci. Rep..

[114] Hanahan D., Weinberg R.A. (2011). Hallmarks of cancer: The next generation. Cell.

[115] Sansregret L., Patterson J.O., Dewhurst S., Lopez-Garcia C., Koch A., McGranahan N., Chao W.C.H., Barry D.J., Rowan A., Instrell R. (2017). APC/C dysfunction limits excessive cancer chromosomal instability. Cancer Discov..

[116] Xin Y., Ning S., Zhang L., Cui M. (2018). CDC27 facilitates gastric cancer cell proliferation, invasion and metastasis via twist-induced epithelial-mesenchymal transition. Cell Physiol. Biochem..

[117] Pawar S.A., Sarkar T.R., Balamurugan K., Sharan S., Wang J., Zhang Y., Dowdy S.F., Huang A.M., Sterneck E. (2010). C/EBPδ targets cyclin D1 for proteasome-mediated degradation via induction of CDC27/APC3 expression. Proc. Natl. Acad. Sci. USA.

[118] Schrock M.S., Stromberg B.R., Scarberry L., Summers M.K. (2020). APC/C ubiquitin ligase: Functions and mechanisms in tumorigenesis. Semin. Cancer Biol..

[119] Fujita T., Liu W., Doihara H., Date H., Wan Y. (2008). Dissection of the APC^Cdh1^-Skp2 cascade in breast cancer. Clin. Cancer Res..

[120] Bonaiuti P., Chiroli E., Gross F., Corno A., Vernieri C., Stefl M., Cosentino Lagomarsino M., Knop M., Ciliberto A. (2018). Cells escape an operational mitotic checkpoint through a stochastic process. Curr. Biol..

[121] Manchado E., Guillamot M., de Carcer G., Eguren M., Trickey M., Garcia-Higuera I., Moreno S., Yamano H., Canamero M., Malumbres M. (2010). Targeting mitotic exit leads to tumor regression in vivo: Modulation by Cdk1, Mastl, and the PP2A/B55α,δ phosphatase. Cancer Cell.

[122] Schvartzman J.M., Sotillo R., Benezra R. (2010). Mitotic chromosomal instability and cancer: Mouse modelling of the human disease. Nat. Rev. Cancer.

[123] Gutgemann I., Lehman N.L., Jackson P.K., Longacre T.A. (2008). Emi1 protein accumulation implicates misregulation of the anaphase promoting complex/cyclosome pathway in ovarian clear cell carcinoma. Mod. Pathol..

[124] Kato T., Daigo Y., Aragaki M., Ishikawa K., Sato M., Kondo S., Kaji M. (2011). Overexpression of MAD2 predicts clinical outcome in primary lung cancer patients. Lung Cancer.

[125] Pernicone N., Peretz L., Grinshpon S., Listovsky T. (2020). MDA-MB-157 cell line presents high levels of MAD2L2 and dysregulated mitosis. Anticancer Res..

[126] Christopoulou L., Moore J.D., Tyler-Smith C. (2003). Over-expression of wild-type Securin leads to aneuploidy in human cells. Cancer Lett..

[127] Chiarle R. (2021). Solving the chromosome puzzle of aneuploidy in cancer. Genes Dev..

[128] Karra H., Pitkänen R., Nykänen M., Talvinen K., Kuopio T., Söderström M., Kronqvist P. (2012). Securin predicts aneuploidy and survival in breast cancer. Histopathology.

[129] Karra H., Repo H., Ahonen I., Löyttyniemi E., Pitkänen R., Lintunen M., Kuopio T., Söderström M., Kronqvist P. (2014). Cdc20 and securin overexpression predict short-term breast cancer survival. Br. J. Cancer.

[130] Liao G.B., Li X.Z., Zeng S., Liu C., Yang S.M., Yang L., Hu C.J., Bai J.Y. (2018). Regulation of the master regulator FOXM1 in cancer. Cell Commun. Signal..

[131] Ye H., Kelly T.F., Samadani U., Lim L., Rubio S., Overdier D.G., Roebuck K.A., Costa R.H. (1997). Hepatocyte nuclear factor 3/fork head homolog 11 is expressed in proliferating epithelial and mesenchymal cells of embryonic and adult tissues. Mol. Cell Biol..

[132] Wang I.C., Chen Y.J., Hughes D., Petrovic V., Major M.L., Park H.J., Tan Y., Ackerson T., Costa R.H. (2005). Forkhead box M1 regulates the transcriptional network of genes essential for mitotic progression and genes encoding the SCF (Skp2-Cks1) ubiquitin ligase. Mol. Cell Biol..

[133] Wang X., Chen D., Gao J., Long H., Zha H., Zhang A., Shu C., Zhou L., Yang F., Zhu B. (2018). Centromere protein U expression promotes non-small-cell lung cancer cell proliferation through FOXM1 and predicts poor survival. Cancer Manag. Res..

[134] Halasi M., Gartel A.L. (2012). Suppression of FOXM1 sensitizes human cancer cells to cell death induced by DNA-damage. PLoS ONE.

[135] Dai B., Kang S.H., Gong W., Liu M., Aldape K.D., Sawaya R., Huang S. (2007). Aberrant FoxM1B expression increases matrix metalloproteinase-2 transcription and enhances the invasion of glioma cells. Oncogene.

[136] Wen N., Wang Y., Wen L., Zhao S.H., Ai Z.H., Wang Y., Wu B., Lu H.X., Yang H., Liu W.C. (2014). Overexpression of FOXM1 predicts poor prognosis and promotes cancer cell proliferation, migration and invasion in epithelial ovarian cancer. J. Transl. Med..

[137] Wang K., Zhu X., Zhang K., Zhu L., Zhou F. (2016). FoxM1 inhibition enhances chemosensitivity of docetaxel-resistant A549 cells to docetaxel via activation of JNK/mitochondrial pathway. Acta Biochim. Biophys. Sin..

[138] Carmena M., Ruchaud S., Earnshaw W.C. (2009). Making the Auroras glow: Regulation of Aurora A and B kinase function by interacting proteins. Curr. Opin. Cell Biol..

[139] Zhang H., Chen X., Jin Y., Liu B., Zhou L. (2012). Overexpression of Aurora-A promotes laryngeal cancer progression by enhancing invasive ability and chromosomal instability. Eur. Arch. Oto-Rhino-Laryngol..

[140] Li J.J., Weroha S.J., Lingle W.L., Papa D., Salisbury J.L., Li S.A. (2004). Estrogen mediates Aurora-A overexpression, centrosome amplification, chromosomal instability, and breast cancer in female ACI rats. Proc. Natl. Acad. Sci. USA.

[141] Bischoff J.R., Anderson L., Zhu Y., Mossie K., Ng L., Souza B., Schryver B., Flanagan P., Clairvoyant F., Ginther C. (1998). A homologue of *Drosophila* aurora kinase is oncogenic and amplified in human colorectal cancers. EMBO J..

[142] Li D., Zhu J., Firozi P.F., Abbruzzese J.L., Evans D.B., Cleary K., Friess H., Sen S. (2003). Overexpression of oncogenic STK15/BTAK/Aurora A kinase in human pancreatic cancer. Clin. Cancer Res..

[143] Anand S., Penrhyn-Lowe S., Venkitaraman A.R. (2003). AURORA-A amplification overrides the mitotic spindle assembly checkpoint, inducing resistance to Taxol. Cancer Cell.

[144] Sinha D., Duijf P.H.G., Khanna K.K. (2019). Mitotic slippage: An old tale with a new twist. Cell Cycle.

[145] Tomasini R., Mak T.W., Melino G. (2008). The impact of p53 and p73 on aneuploidy and cancer. Trends. Cell Biol..

[146] Katayama H., Wang J., Treekitkarnmongkol W., Kawai H., Sasai K., Zhang H., Wang H., Adams H.P., Jiang S., Chakraborty S.N. (2012). Aurora kinase-A inactivates DNA damage-induced apoptosis and spindle assembly checkpoint response functions of p73. Cancer Cell.

[147] Colicino E.G., Hehnly H. (2018). Regulating a key mitotic regulator, polo-like kinase 1 (PLK1). Cytoskeleton.

[148] Cholewa B.D., Liu X., Ahmad N. (2013). The role of polo-like kinase 1 in carcinogenesis: Cause or consequence?. Cancer Res..

[149] Lens S.M., Voest E.E., Medema R.H. (2010). Shared and separate functions of polo-like kinases and aurora kinases in cancer. Nat. Rev. Cancer.

[150] Knecht R., Oberhauser C., Strebhardt K. (2000). PLK (polo-like kinase), a new prognostic marker for oropharyngeal carcinomas. Int. J. Cancer.

[151] Wolf G., Elez R., Doermer A., Holtrich U., Ackermann H., Stutte H.J., Altmannsberger H.M., Rübsamen-Waigmann H., Strebhardt K. (1997). Prognostic significance of polo-like kinase (PLK) expression in non-small cell lung cancer. Oncogene.

[152] Liu X., Erikson R.L. (2003). Polo-like kinase (Plk)1 depletion induces apoptosis in cancer cells. Proc. Natl. Acad. Sci. USA.

[153] Li Z., Liu J., Li J., Kong Y., Sandusky G., Rao X., Liu Y., Wan J., Liu X. (2017). Polo-like kinase 1 (Plk1) overexpression enhances ionizing radiation-induced cancer formation in mice. J. Biol. Chem..

[154] Gao Y., Zhang B., Wang Y., Shang G. (2018). Cdc20 inhibitor apcin inhibits the growth and invasion of osteosarcoma cells. Oncol. Rep..

[155] De K., Grubb T.M., Zalenski A.A., Pfaff K.E., Pal D., Majumder S., Summers M.K., Venere M. (2019). Hyperphosphorylation of CDH1 in glioblastoma cancer stem cells attenuates APC/C^CDH1^ activity and pharmacologic inhibition of APC/C^CDH1/CDC20^ compromises viability. Mol. Cancer Res..

[156] Lub S., Maes A., Maes K., De Veirman K., De Bruyne E., Menu E., Fostier K., Kassambara A., Moreaux J., Hose D. (2016). Inhibiting the anaphase promoting complex/cyclosome induces a metaphase arrest and cell death in multiple myeloma cells. Oncotarget.

[157] Zhang Y., Xue Y.B., Li H., Qiu D., Wang Z.W., Tan S.S. (2017). Inhibition of cell survival by curcumin is associated with downregulation of cell division cycle 20 (Cdc20) in pancreatic cancer cells. Nutrients.

[158] Cheng S., Castillo V., Sliva D. (2019). CDC20 associated with cancer metastasis and novel mushroomderived CDC20 inhibitors with antimetastatic activity. Int. J. Oncol..

[159] Kudlow B.A., Kennedy B.K., Monnat R.J. (2007). Werner and Hutchinson-Gilford Progeria Syndromes: Mechanistic basis of human progeroid diseases. Nat. Rev. Mol. Cell Biol..

[160] Gonzalo S., Kreienkamp R., Askjaer P. (2017). Hutchinson-Gilford Progeria Syndrome: A premature aging disease caused by LMNA gene mutations. Ageing Res. Rev..

